# Analysis of Scientific Research Driving Microalgae Market Opportunities in Europe

**DOI:** 10.3390/md18050264

**Published:** 2020-05-18

**Authors:** Judith Rumin, Elodie Nicolau, Raimundo Gonçalves de Oliveira Junior, Claudio Fuentes-Grünewald, Laurent Picot

**Affiliations:** 1 La Rochelle Université, UMRi CNRS 7266 LIENSs, Avenue Crépeau, 17042 La Rochelle, France; judith.rumin@univ-larochelle.fr (J.R.); oliveira.farma.junior@gmail.com (R.G.d.O.J.); 2 IFREMER, Laboratoire BRM/PBA, Rue de l’Ile d’Yeu, 44311 Nantes, France; Elodie.Nicolau@ifremer.fr; 3 Department of Biosciences, Swansea University, Singleton Park, Swansea, Wales SA2 8PP, UK; c.fuentesgrunewald@swansea.ac.uk

**Keywords:** bibliometric, microalgae, biofertilizers, bioplastics, biotechnology, cosmetics, Europe, food, GMO, market, nutraceuticals, pharmaceuticals, research

## Abstract

A bibliographic database of scientific papers published by authors affiliated to research institutions worldwide, especially focused in Europe and in the European Atlantic Area, and containing the keywords “microalga(e)” or “phytoplankton” was built. A corpus of 79,020 publications was obtained and analyzed using the Orbit Intellixir software to characterize the research trends related to microalgae markets, markets opportunities and technologies that could have important impacts on markets evolution. Six major markets opportunities, the production of *biofuels*, *bioplastics*, *biofertilizers, nutraceuticals*, *pharmaceuticals* and *cosmetics*, and two fast-evolving technological domains driving markets evolution, *microalgae harvesting and extraction technologies* and *production of genetically modified (GM-)microalgae*, were highlighted. We here present an advanced analysis of these research domains to give an updated overview of scientific concepts driving microalgae markets.

## 1. Introduction

The use of microalgae biomass as an alternative and innovative source to produce food, feed ingredients, cosmetics, biopharmaceuticals, nutraceuticals and renewable energy has been well established and documented [1,2,3,4,5,6,7,8,9]. With an estimated number of 30,000–1,000,000 species, microalgae represent an exceptional natural resource to explore for the 21st century that combines numerous advantages, such as original metabolisms and chemical compositions, fast growth, high photosynthetic efficiency, non-competition for farmlands and tolerance to wastewaters during cultivation. However, despite these major advantages, scientific, technological, legislative, administrative and marketing gaps are barriers still exist that limit the commercial development of microalgae and microalgae-related products. In December 2018, as part of the Interreg EnhanceMicroalgae EAPA 338_2016 project [10], a workshop was organized in la Rochelle, France, to discuss and identify with 12 European microalgae experts the market opportunities arising from European research on microalgae and cyanobacteria, and define gaps and barriers limiting the industrial and markets developments. These European microalgae experts were selected as a representative panel of researchers and industrials with a high knowledge and experience in microalgae field, technologies and markets.

The first statement was that Europe presents a high potential for microalgae research, innovation and industrial development, bringing together a critical number of expert researchers, companies and technological platforms. Accordingly, a bibliometric analysis of microalgae research revealed that the European scientific community published 26,137 papers in the field of microalgae and phytoplankton until 2019 [11]. (This bibliometric analysis used 26,137 European references available in Scopus, these references are available on demand to the corresponding author.) Most of the papers of that analysis were focused on the function of microalgae in the environment (8,962 publications) and their possible use to produce food and chemicals (4275 and 4271 publications, respectively). Going deeper into the research topics analysis, papers were found to mainly focus on pigments (1861), proteins (1847), feed (1818), drug (1474), biofuel (1014) and biotechnologies (892), but also on recent niche markets in the fields of biostimulants (13), bioplastics (14), vaccines (16), biofertilizers (22) and nanotechnology (59) [11]. Accordingly, European experts highlighted six major market opportunities for microalgae: the production of bioplastics, biofertilizers, nutraceuticals, pharmaceuticals, cosmetics and biofuels. Two research and development sectors in link with industrial technologies were also emphasized as driving the microalgae markets evolution: the transformation of microalgae to obtain genetically modified (GM-)strains and microalgae harvesting and extraction technologies to improve production yields and add value to microalgae-related products.

In this paper, we propose an advanced analysis of European scientific publications to highlight research trends driving microalgae markets, characterize markets opportunities for microalgae and identify emerging microalgae markets in Europe. The main research concepts, concept networks, emerging concepts and volume of publications over years for major markets and related technologies are detailed. This work complements an in-depth global analysis of microalgae research in the world, Europe and the European Atlantic Area, recently published in this journal [11].

## 2. Results

### 2.1. Technological Research Driving Microalgae Markets

#### 2.1.1. Genetically Modified Microalgae (GM-Microalgae)

**Figure 1:**
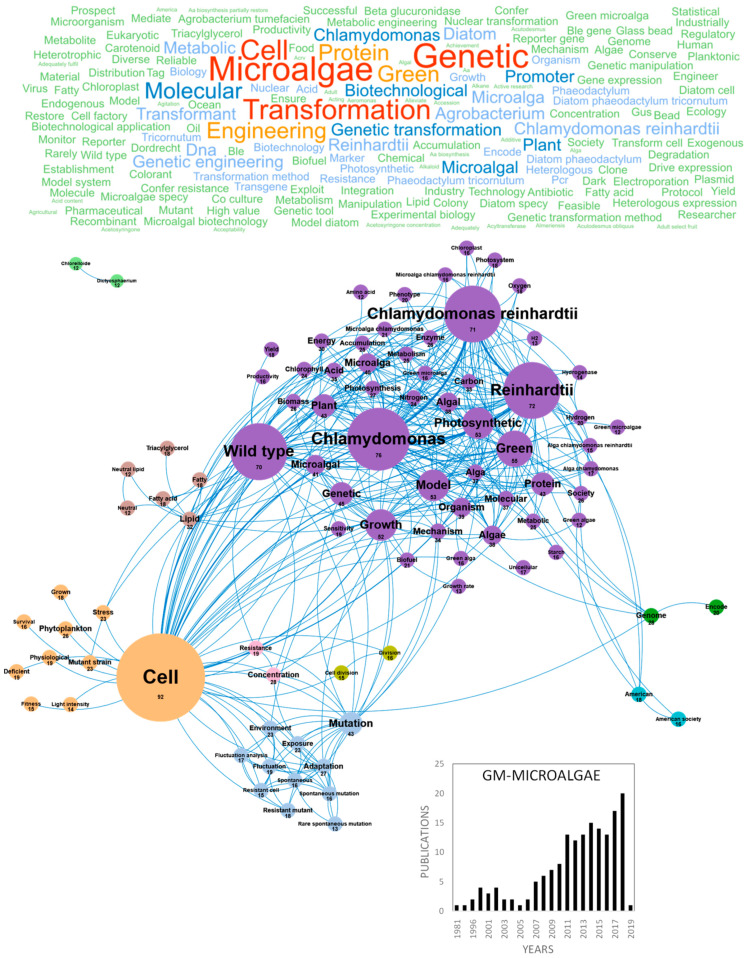
Main concepts, concepts network and annual number of European scientific publications related to genetically modified microalgae (GM-microalgae) in 166 publications (400 links; 28 coocc; 12 occ; and 9 clusters).

Technical advances in the genetic manipulation of microorganisms and genome sequencing of microalgae have stimulated the development and use of GM-microalgae for biotechnological applications [12]. This emerging research domain, with strong ethical and regulatory framework, is mostly dedicated to the production of GM-strains producing high added value compounds for health or biotechnological applications such as recombinant proteins (vaccines and hormones) or biofuel. The production of GM-strains, which requires a strictly controlled environment complying with European regulations [13], is completed with mutations-selection strategies to generate strains with desirable commercial traits such as tolerance to excess light and heat stress, resistance to herbivore/pathogen, capacity to outcompete opportunistic organisms or to overexpress biosynthetic pathways [12]. Publications studying or reviewing GM-microalgae are regularly produced in Europe. Figure 1 highlights the main concepts and concepts networks arising from these papers. Microalgae transformation using genetic engineering has generated interest since 1981 but did not drive more than four publications/year in Europe until 2017. Following 2017, a faster development of domesticated strains and acceleration of genome sequencing led to more than 20 publications/year (Figure 1). France, Germany and Spain were the main countries publishing in this field with 52, 44 and 36 publications, respectively (Figure 10a). The main concepts emerging from this European database on GM-microalgae were *microalgae*, *transformation* and *genetic*. These concepts are linked to the *productivity* of microalgae molecules and are very connected, as shown in Figure 1. This figure also highlights strategies to improve a character of interest (high *growth rate*, *overaccumulation*, *deficiency*, *fitness*, *survival*, *deficient*, *stress* and *light intensity*). The increase in *yield* of molecules of interest targets *hydrogen/hydrogenase, starch, amino acid, acid* or *resistance* to biotic or abiotic stress. The cluster including *lipid* and *fatty acid* is isolated because of the large number of publications aiming at improving the productivity of microalgal strains to produce biofuel. The full realization of this potential in microalgae also requires research and progress in the cultivation and growth techniques either by understanding and increasing the capacity of the photosynthetic apparatus as highlighted in the *photosystem*, *chloroplast, photosynthetic*, *photosynthesis* and *energy* concepts or by optimization of cultivation in photobioreactor to maximize the efficiency of solar energy conversion into biomass and bio-products to achieve a profitable product [12,14]. The genetic tools required for these improvements were the genetic manipulation of species (*mutation*, *mutant strains*, *molecular*, *resistant mutant* and *cell*), the analysis of metabolic pathways (*model*, *mechanism* and *metabolic*) and genome editing (*genome* and *encode*). A large part of the molecular genetic studies was carried out in one specific species whose genomes have been sequenced such as the model *green* species *Chlamydomonas reinhardtii* by comparing mutated strains to the *wild type*. More recently, the concepts that have emerged from this GM-microalgae research domain are *acid*, *concentration*, *heterologous expression*, *microorganism*, *protocol*, *transformation condition*, *acid content*, *Acutodesmus obliquus*, *acyltransferase*, *Agrobacterium tumefaciens*, *biodiesel*, *biomass*, *Boraginaceae*, *Bzip14*, *cell*, *carbon* and *carbon reallocation*. The development of new molecular tools such as the *CRISPR/Cas9* technology will probably stimulate research, funding and evolution of the regulation to achieve economic viability and support innovative applications in the field of GM-microalgae biotechnology in Europe. It should be noted that all this GM-microalgae work has been done at lab scale and in strictly biosafety GM conditions because of the strict EU regulatory issues on GM species. As far as the authors know, no GM microalgae species has been grown in Europe neither at pilot scale (<1000 L in closed system such as photobioreactors) nor at industrial scale.

#### 2.1.2. Microalgae Harvesting and Extraction

**Figure 2:**
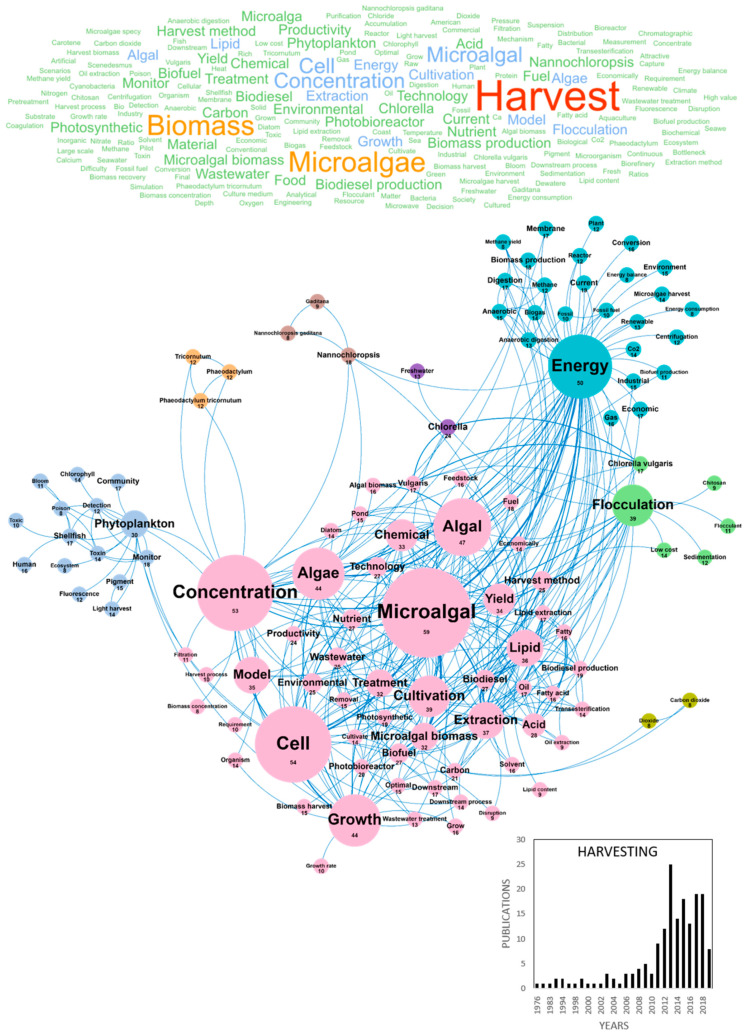
Main concepts, concepts network and annual number of European scientific publications related to the microalgae harvesting in 175 publications (400 links; 7 coocc; 8 occ; and 8 clusters).

Despite the potential of high-added value molecules from microalgae, current production costs still constitute a limiting barrier to the microalgae economic viability and introduction to markets in Europe. Technological improvements are thus needed to reach a cost-effective large-scale production of microalgae-based products that could stimulate the creation of a dense microalgae industrial sector. Among these technologies, harvesting and drying of microalgae biomass have been identified as key bottlenecks as they contribute to one third of the final biomass cost [12]. In European papers, it can be shown that microalgae biomass is mostly harvested by chemical coagulation/flocculation, auto and bioflocculation, gravity sedimentation, flotation, electrical base processes, filtration and centrifugation [15]. In our database, 175 papers dealt with microalgae harvesting processes and the volume of publications was about 20 publications per year since 2012 (Figure 2). The main concepts contained in these publications were *harvest*, *microalgae/microalgal*, *biomass*, *cell*, *concentration*, *energy*, *algal/algae*, *growth*, *cultivation*, *flocculation*, *extraction*, *lipid* and *model*. The *flocculation* cluster in green, linked to *Chlorella vulgaris* and *low-cost* concepts, highlights the dominance of this harvesting method in recent years. Among the most cited publications, harvesting methods studied were flocculation, bio-flocculation, electro-coagulation-flocculation and microfiltration membranes (Table 1). The main country publishing on microalgae harvesting was Spain with a total of 52 publications, followed by Italy, Belgium, Germany and France with 22, 20, 20 and 20 publications, respectively (Table 2). Emerging concepts identified in the two last years on microalgae harvesting publications are presented in Table 3. No innovative concepts emerge from this list, but a large part of studies deal with the assessment of new flocculants such as chitosan (Figure 2), *aluminum* chloride or *copper* sulfate [16,17,18,19,20] (Table 3). Additionally, it should be noted that innovative microfluidics harvesting systems are developed by companies. Many publications discussing microalgae harvesting had a link with the production of lipids, as demonstrated by the connection with the *C16* (Palmitic acid) and *C18* (Stearic acid) emerging concepts.

**Figure 3:**
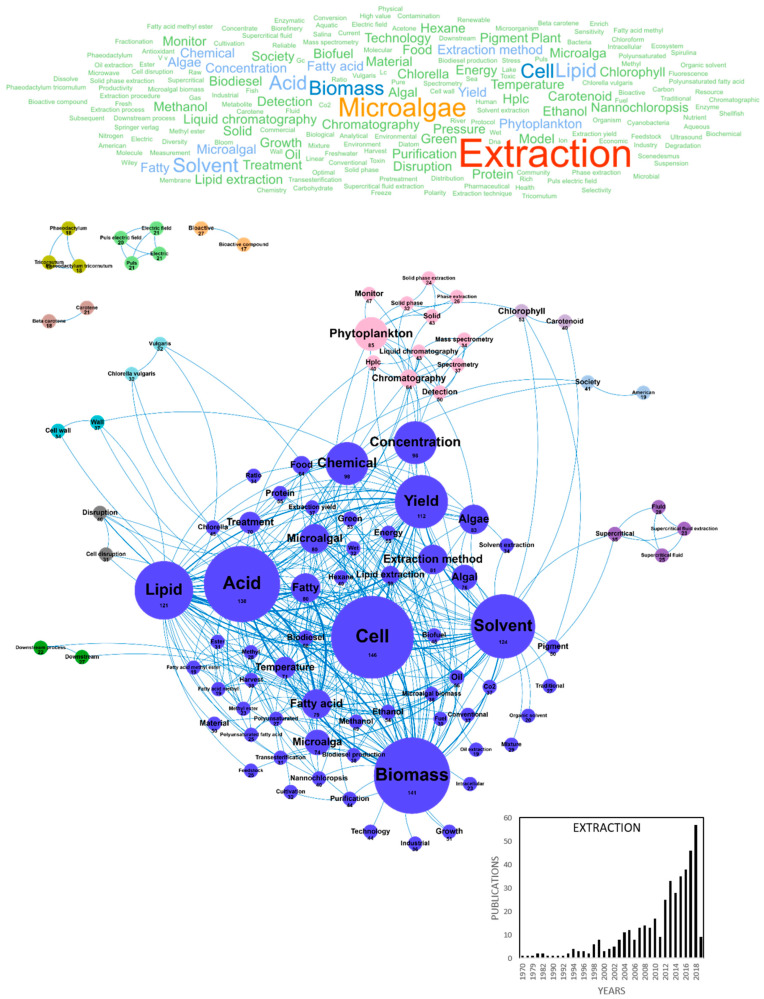
Main concepts, concepts network and annual number of European scientific publications related microalgae extraction in 427 publications (400 links; 17 coocc; 17 occ; and 13 clusters).

Improving the production of high-added value molecules from microalgae and reaching a cost-effective production also depend on developing innovative extraction processes such as supercritical CO_2_ extraction, membrane micro-ultra-nanofiltration/fractionation or nannobubbles technology [34]. In our database, 427 publications studied this challenge, with a production of about 25 publications per year since 2012 reaching 57 publications in 2018 (Figure 3). The main concepts found in these publications were *extraction*, *microalgae/microalgal*, *cell*, *biomass*, *acid*, *solvent*, *lipid*, *yield*, *chemical*, *concentration*, *phytoplankton*, *algae/algal*, *extraction method* and *fatty acid*. Most of these concepts constituted major clusters, dominated by lipid extraction from microalgae with solvents (*acid*, *lipid*, *lipid extraction*, *solvent*, *ethanol*, *methanol*, *hexane*, *transesterification* and *fatty methyl*). However, the *supercritical fluid extraction* cluster in purple highlights the recent interest for this innovative extraction method. Among the most cited extraction processes were also described ultrasound-assisted extraction, microwaves-assisted extraction and hydrothermal treatment (Table 4). Most publications dealing with microalgae extraction in collaboration with the AA came from Spain with 120 publications, followed by France, Italy and Germany with 88, 63 and 62 publications, respectively (Table 5). Some emerging concepts identified since 2017 about microalgae extraction are listed in Table 6. Beyond limiting downstream processes for an efficient extraction process, the scientific community was mostly interested in *optimizing green* extraction techniques, using *solvents-free*, *clean*, *scalable*, *economic* and *sustainable* extracting processes (Figure 3) (Table 4) [35,36].

### 2.2. Top 6 Market Opportunities for Microalgae Research

#### 2.2.1. Microalgae-Based Biofuels

**Figure 4:**
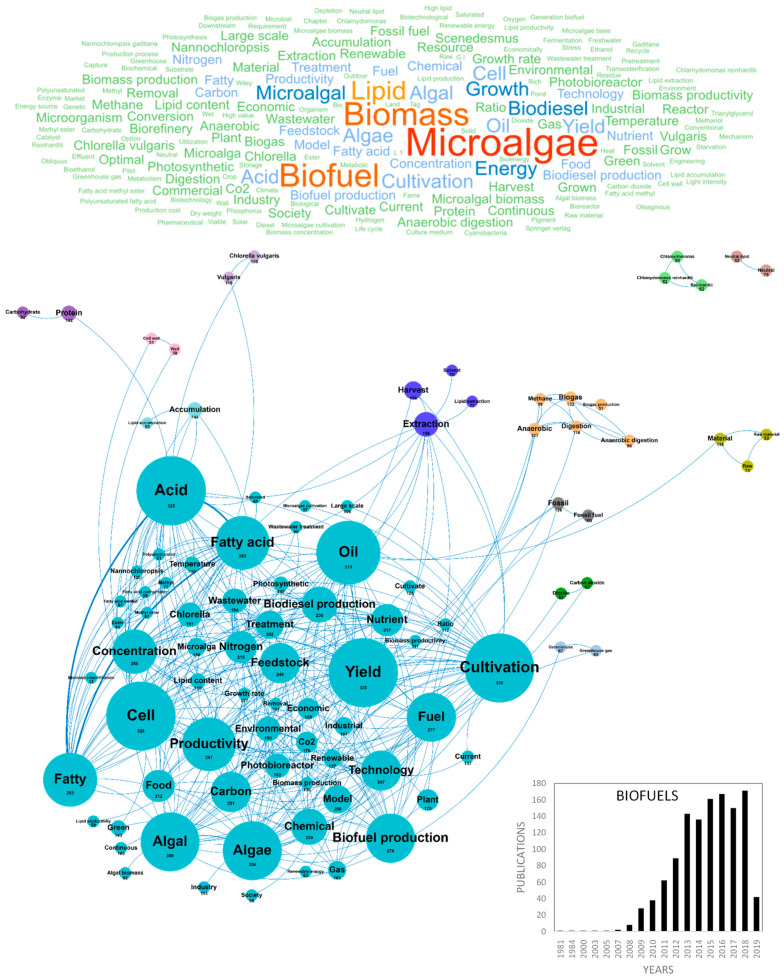
Main concepts, concepts network and annual number of the European scientific publications related to the biofuels field in 1202 publications (400 links; 48 coocc; 48 occ; and 13 clusters).

One of the major concerns of modern societies is the exhaustion of conventional “fossil” resources with the increase of human population, expected to reach a total of 9 billion people by 2050 [47]. Microalgae have received a great deal of interest as potential biofuel producers in response to energy crisis, global warming and climate changes [48]. It is indeed estimated that they could provide up to 25% of the required energy [15,49]. Ecofriendly, nontoxic microalgae-based oils can be used as raw material for the production of biodiesel, biomethane, bioethanol, biohydrogen and biobutanol, in a sustainable cycle including atmospheric CO_2_ fixation. The first investigations on microalgal bio-energy started in the 1950s and countries in Asia, Europe and America have now started the industrialization of biofuels production from microalgae biomass [50]. In Europe, 1202 scientific publications related to this topic were produced until 2019 and this number should keep on increasing dramatically in the near future as microalgae-based biofuels are still not economically profitable [48]. The challenges to overcome are related to production scalability, operational stability and associated costs compared to other energy sources. Most studies dedicated to the production of biofuels by microalgae focused on improvements of cultivation, harvesting, extraction, biorefinery and conversion methods [51]. In Europe, numerous in-depth reviews of the literature dealt with microalgae-based biofuels and 152 articles containing both the words “review” and “biofuels” were found in our database. Figure 4 shows the word clouds of the main concepts contained in the European scientific publications, networks and clusters established between these concepts and the temporal production of these publications. The scientific production on biofuels increased considerably in the early 2010s from 8 publications/year in 2008 to 143 publications/year in 2013. Since then, the number of papers stabilized with approximately 160 publications per year. Spain, Italy and France were the top three countries publishing in the field of microalgae-based biofuels with around 200 publications (229, 200 and 191, respectively). The main concepts that emerged from this biofuels European database were *microalgae*, *biomass*, *biofuel*, *lipid*, *growth*, *biodiesel* and *energy* (Figure 4). After dismissing these ubiquitous concepts, 13 clusters could be highlighted. The main cluster in blue characterizes the concepts of the *lipid productivity* of the *cell*. One of the major scientific challenge for biofuels (specifically biodiesel) production is indeed to optimize the lipid production yield of microalgae. The efficiency objectives are clearly identified by the *productivity, feedstock, production* and *yields* concepts as well as the *fatty acid, algal, cell* and *oil* concepts and focused on economic viability of biodiesel production systems (*industrial*, *industry*, *economic* and *large-scale)*. Microalgae contain a rich diversity of carbon compounds that can be transformed into biofuels but lipids are the most promising class of biomolecules, on which efforts have been focused to date, as shown in Figure 4. Most microalgae species are favorable for biodiesel production because of their high lipids contents (50–70%) and some species such as *B. braunii* can reach lipids contents up to 80% [3,52]. Another first line of improvement for the near future is related to the scalability and costs of microalgae *cultivation* [51]. The aim is to exploit the biodiversity and physiology of microalgae and optimize the culture parameters in *photobioreactors* for the production and *accumulation* of biomolecules (lipids, pigments, carbohydrates and hydrocarbons) precursors to biofuels. Biomass production yields highly depend on algae strain, CO_2_ supply and light regime [53] and it is estimated that microalgae have the potential to transform 9–10% of solar energy (average sunlight irradiance) into biomass with a theoretical yield of 77 g/biomass/m^2^/day, corresponding to 280 ton/ha/year [54,55]. However, this yield is lower at larger scale cultivation and in photobioreactors because of light absorption by biomass and culture equipment [56]. Applied research tends to solve the obstacles of light intensity loss by proper shaking and mixing of the culture in the bioreactor, allowing the uniform distribution of light energy and maximum light energy conversion to biomass [50]. Biomass production efficiency also highly depends on nutrients feeding [53]. Only in the last decade, the problem of nutrients demand in industrial microalgae sustainable cultivation became a matter of concern in the scientific community, with a special concern for Nitrogen (N) and Phosphorus (P) [57,58], two nutrients required to achieve significant biomass. As microalgal biomass contains about three times the amount of nutrients present in terrestrial plants, competition among energy, bio-products and food production might be shifted from lands to fertilizers issues [58]. The integration of microalgae cultivation with *wastewater* treatment in biofuels production is one of the most promising solutions and numerous European publications contain the *nutrient*, *nitrogen*, *carbon*, *ratio*, *phosphorus* and *removal* concepts. Recently, efforts were reported to use municipal, agricultural and industrial wastewater sources to recover these valuable nutrients [59,60,61,62,63] in a logic of *nutrient removal* and *bioremediation*. It was reported that 1 kg of algal biomass can fix 1.83 kg of CO_2_ and also that some species could use SOx and NOx as nutrient flows along with CO_2_ [50,64]. High nutrient removal efficiencies and high productivities in microalgal biomass, lipid and biodiesel were achieved simultaneously using piggery wastewater to cultivate microalgae in photobioreactors [48]. However, the nutrients bio-availability, their utilization efficiency and possible toxicity need to be carefully assessed [58]. Research also tends to use flue gas point sources to maximize carbon capture capacity, while sustaining the stability of algal culture in function of pollutants/nutrients concentrations, temperature and pH variations. Coupling microalgae culture with flue gases depollution could minimize depollution costs, gases transport and storage costs while producing biomass for biodiesel extraction [51]. These studies highlight the growing interest of Europe for greener and sustainable options to produce biofuels at industrial scale and valorize industrial effluents, evidenced by the *environmental*, *greenhouse gas* and *green* concepts (Figure 4). Metabolic engineering of algae is also an active research domain aiming to identify and control the key molecular and physiological mechanisms involved in the production of biofuel precursors. Metabolic modeling, highlighted by the *model* concept, could soon become an essential decision support tool to identify the main factors modulating lipids metabolism and explore microalgal growth and productivity using simulation platforms [65]. Most scientific studies on biofuels production were performed with the *Chlorella* and *Nannochloropsis* genera and related to the *Chlorella*, *cultivation* and *ratio* concepts while *Chlamydomonas* sp. was also very studied and associated to the *starch accumulation* and *proof of concept* cluster (Figure 4). Beside microalgae cultivation, harvesting the biomass in suspension cultures also participates to the high cost of biofuels production, as highlighted by the *extraction* and *harvest* clusters in deep blue (Figure 4). Harvesting process contributes considerably in the overall biomass production cost as it usually accounts for about up to 30% of the total biomass cost [15]. The challenges in harvesting and extraction of microalgae are mainly related to the energy input, especially for concentrating and drying diluted algae cultures. The major methods currently used include flocculation, centrifugation, filtration, gravity sedimentation, electrophoresis, flotation and combinations of these [51]. The cluster including *carbohydrates* and *proteins* is isolated because apart from extracted lipids converted into biodiesel, carbohydrates such as glycogen, starch, agar and cellulose can be converted to fermentable sugars for bioethanol production [51,66]. Research in Europe also focuses on the conversion of the microalgae biomass into biomethane and biohydrogen by anaerobic digestion (*biogas, biomethane, digestion* and *anaerobic*). As for biodiesel, the development of these biofuels will require effort regarding the cultivation and production of carbohydrate-rich biomass, biomass processing (dewatering and harvesting) and improvement of fermentation yield. The concepts that emerged recently from the microalgae biofuels research domain were *biomass cultivate*, *energy sector*, *fermentative hydrogen*, *semi continuous mode*, *simulation tool*, *suitable fatty acid*, *anaerobic co digestion*, *auxenochlorella*, *capture carbon*, *cascade* and *copper*. It should also be noted that some recent works aim to extract lipidic biofuels precursors without destroying algal cells. As a conclusion, after several decades of research, it can be considered that algal biofuels production has reached a high technology readiness level (TRL 6), but that competitivity improvements in microalgae growth techniques, harvesting technologies and genetic engineering [50] are still required to make it commercially viable at high scale. A better valorization by using biorefinery approach of microalgae biomass process in order to have several by-products and an efficient and sustainable coupling of microalgae growth with wastewater treatment should help improve the competitivity of microalgae-based biofuels [48].

#### 2.2.2. Microalgae-Based Bioplastics

**Figure 5:**
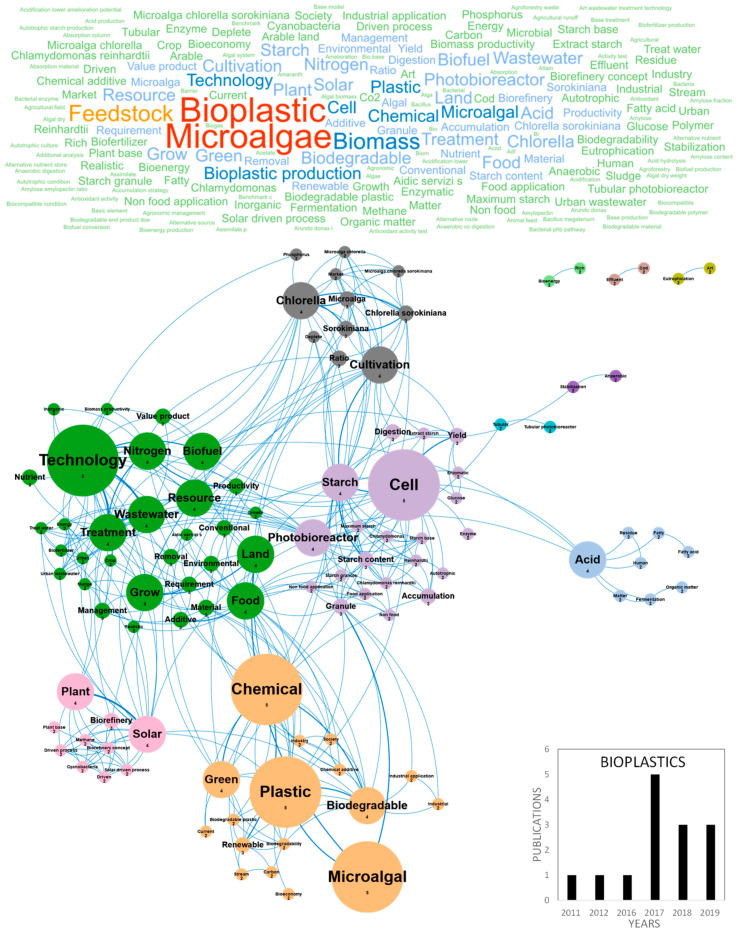
Main concepts, concepts network and annual number of the European scientific publications related to the bioplastic field in 14 publications (400 links; 2 coocc; 22 occ; and 11 clusters).

The huge accumulation of plastic waste in the world is driving international demand and stimulating research for the production of renewable and biodegradable plastics [67]. Particularly, the ubiquitous and persistent presence of microplastics in the oceans represents a serious threat for the marine environment, affecting trophic networks from microalgae to superior predators. A direct toxicity of microplastics on microalgae has been demonstrated, even at very low concentrations (in the ppm range) and can be partly explained by their adsorption and aggregation on cells inducing chlorophyll and photosynthesis reduction, oxidative stress, morphological changes and growth inhibition, eventually impacting all marine food webs [68,69]. In this context, microalgae biomass is considered an innovative eco-friendly resource for producing bioplastics, mainly using polyhydroxyalkanoates (PHA) as a raw material. It can be used directly or as a feedstock for secondary processes with existing infrastructures and processes [70]. Publications reviewing and studying the different kinds of microalgae-based bioplastics, their chemical properties, their markets and the key barriers limiting their development are currently emerging in the literature. Figure 5 shows the word clouds of the main concepts contained in the European scientific publications, networks and clusters established between these concepts and the temporal production of these publications. Only 3–5 papers were published per year on the topic of bioplastics since 2017 and the number is growing, with three papers already published in the first two months of 2019. Germany and Italy were the main countries publishing microalgae-based bioplastics studies with five and four publications, respectively (Figure 10b). The main concepts emerging from this European database on bioplastics were *microalgae*, *bioplastic*, *feedstock*, *biomass* and *bioplastic production* (Figure 5). By dismissing these ubiquitous concepts, a network of 11 differentiated clusters was created. A first descriptive cluster in orange characterizes the eco-friendly advantage of microalgae in the field of bioplastics by bringing together the *chemical*, *plastic*, *green*, *biodegradable*, *renewable* and *bioeconomy* concepts. Publications also highlighted the advantages of bioplastics for *industrial application* and compatibility with the establishment of a circular *bioeconomy* which is beginning to develop [67,71]. Among the expanding microalgae circular bioeconomy trend in which companies are also involved for bioplastics, this cluster underlines Europe’s current concern for ecology, the need for new greener options for the production of chemicals and polymer materials, combined to the limitation of arable land use, contribution to CO_2_ capture and waste/byproducts use [71]. A second cluster in green highlights the technical description of concepts related to microalgae cultivation for bioplastics including *technology*, *wastewater*, *nutrient*, *treatment* and *productivity*. Nutrient management in microalgae culture to produce bioplastics is a key point linked to the use of wastewater and many studies dedicated to microalgae culture for the production of *biofuel* also discussed *wastewater* treatment [62]. The *photobioreactor* concept highlighted studies dedicated to the physiological orientations of microalgae metabolism to produce bioplastics and made the link with the purple cluster related to starch accumulation in microalgae, containing the *cell*, *starch*, *cultivation* and *accumulation* concepts. When microalgae cells are placed in stress conditions in the culture medium, particularly in nutrients depletion conditions (nitrogen, sulfur or phosphate), they accumulate biopolymers [72,73,74] such as polysaccharides, including starch [75,76], lipids [6,77], proteins [78], pigments [79] and other metabolites. Research also aims at giving a proof-of-concept for the production of microalgae-based bioplastics. A recent study has shown that some strains of *Chlamydomonas reinhardtii* can produce up to 49% w/w of starch in a sulfur-deprived medium and demonstrated the feasibility of up-scaling microalgae starch to small-scale production in 30 L tubular photobioreactor [80]. The starch-rich biomass presented interesting plasticization capacity and in-depth analysis of conditions and protocols of microalgae plasticization were discussed [61]. Another cluster in grey highlights which microalgae species were studied for the production of bioplastics. *Chlorella sorokiniana* was the most cultivated species in these studies and the following concepts were dominant: *Chlorella*, *cultivation* and *ratio. Chlamydomonas* sp. was also very studied and associated to the cluster dealing with *starch accumulation* and *proof of concept*. An isolated cluster in pink highlights the *biorefinery*, *solar* and *methane* concepts. One way to connect the microalgae biomass feedstock into the existing petrochemical-based plastics industry is to convert biomass to methane through fermentation. A wide range of chemical reactions have thus been developed to further convert methane into precursors for bioplastics production. Methane can be used to produce polyhydroxyalkanoates or PHAs, lactic acid (precursor to polylactic acid or PLA), ethanol (precursor to bio-polyethylene or bio-PE and bio-poly vinyl chloride or bio-PVC) and proteins (precursor to proteins-based polymers). Finally, another cluster could be differentiated around the concepts of *acid* and *fermentation* that highlighted the importance of PLA in the current global economy. Polylactic acid (PLA) is a thermoplastic aliphatic polyester derived from renewable biomass and typically obtained from fermented plant starch such as corn, cassava, sugarcane or sugar beet pulp. In 2010, PLA was the second most consumed bioplastic in the world. [67]. The emerging concepts associated to the bioplastic database were *acid*, *biorefinery*, *cultivation*, *digestion*, *accumulation*, *anaerobic*, *bioeconomy*, *biorefinery concept*, *cyanobacteria*, *driven*, *driven process*, *extract starch*, *fatty acid*, *human*, *methane*, *residue*, *solar driven process* and *stabilization*. These were the same concepts that appeared in the figure of network concepts because of the youth of this market with one publication per year in 2011, 2012 and 2016 (Figure 5). It can however be noted that emergent research is developed on cyanobacteria, that constitute an immense reservoir of poorly studied species for the production of bioplastics; some examples are from *Nostoc* genus which produce interesting PHA concentrations. Finally, the authors of these studies stated that the production of durable bioplastics from microalgae for packaging, biomedical or leisure applications may be a long-term challenge [80].

#### 2.2.3. Microalgae-Based Biofertilizers

**Figure 6:**
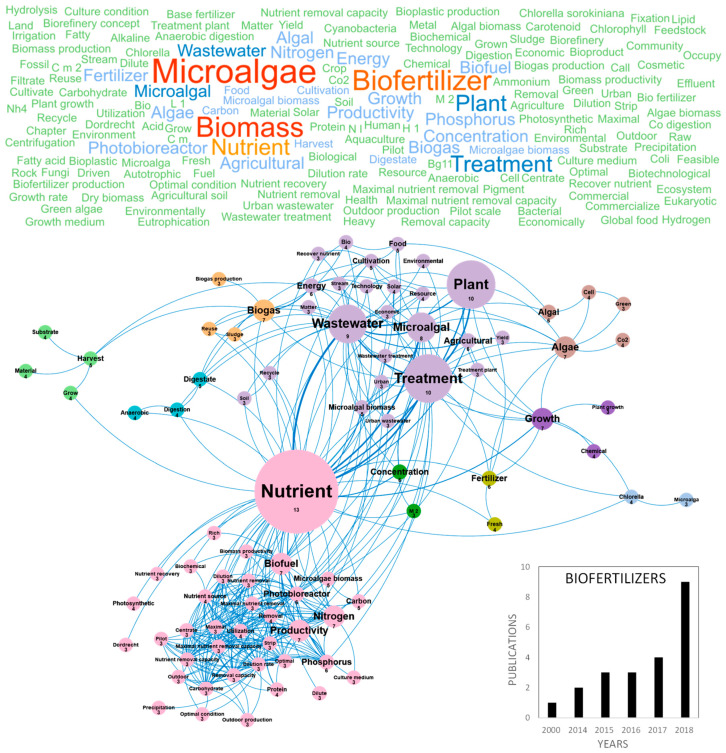
Main concepts, concepts network and annual number of the European scientific publications related to the biofertilizer field in 22 publications (400 links; 3 coocc; 3 occ; and 10 clusters).

The potential of algae-based biofertilizers and biostimulants in agriculture has been increasingly recognized during the recent years. Biofertilizers can provide cost-effective alternatives to synthetic products and renewable and eco-friendly solutions for the future of agriculture. They exhibit a high potential for biostimulation and improvement of crop productivity, improvement of soil fertility and protection against abiotic stresses [81]. The global increase of population and water consumption, the decrease of water supply in some geographic areas, terrestrial and underground water pollution and climate change are currently generating a water crisis that stimulates the research on plant nutrition, wastewater recycling and management of nutrient cycles. In this context, the eco-friendly production of microalgae-based biofertilizers appears as a realistic alternative for the environmental management of wastewater combined to the supply of nutrients for crops [82]. The number of publications that review and study microalgae-based biofertilizers has increased in recent years and the two main countries publishing in this topic were Spain and Italy (Figure 10c). Figure 6 shows the word clouds of the main concepts contained in microalgae scientific publications dealing with biofertilizers, networks and clusters established between these concepts and the temporal production of these publications. The number of publications increased linearly since 2014 reaching nine publications per year in Europe in 2018. The main concepts that emerged from this European database were *microalgae*, *biofertilizer* and *biomass* (Figure 6). After dismissing these ubiquitous concepts, 10 clusters could be highlighted. The *nutrient* concept in pink, located at the center of the biofertilizer concepts network, is linked to the cluster including *productivity*, *nitrogen*, *phosphorus*, *photobioreactor*, *optimal condition* and *biofuel*. Nitrogen, phosphorus and potassium (NPK) are indeed the top three primary nutrients used as commercial fertilizers [83]. They are also the top three nutrients studied for microalgae growth, to produce *microalgae biomass* that can be transformed into biofuels or biofertilizers. *Nutrient removal* yields by microalgae were studied with the aim to achieve an integrated nutrient management (INM) fulfilling environmental regulation. Optimization of microalgae growth conditions to obtain efficient nutrients removals at industrial scale was performed by designing new photobioreactors, such as semi-closed 30 m^3^ horizontal photobioreactors [82]. The treatment of urban or industrial waste waster by microalgae is an option at industrial scale in USA and Europe and it was also considered from the technological and energetical points of view, in an objective of agricultural valorization, as evidenced by the purple cluster grouping the *wastewater*, *agricultural*, *urban*, *technology* and *energy* concepts. Minor clusters such as *biogas* in orange, including the *biogas*, *reuse* and *sludge* concepts or that of *digestion* in blue-green including the *digestate*, *digestion* and *anaerobic* concepts, highlight the interest of the microalgae wastewater bioremediation to produce biomass that can be valorized into ethanol, biohydrogen and biogas [71]. Studies were also conducted to identify the microalgae species combining the best bio-remediation and biofertilizing capacities. For example, *Chlorella* sp. was shown to present high phytostimulating activities on wheat (30% increase in plant length) and proposed as a possible substitute to chemical fertilizers to increase soil fertility [82]. *Nannochloropsis* sp. was also identified as a sustainable biofertilizer increasing the sugar and carotenoid contents in tomato [84]. The blue cluster containing the *Chlorella* concept shows that the majority of scientific publications dealing with biofertilizers used *Chlorella* sp. as a model species [85]. The scientific community was also interested by *Acutodesmus* sp., *Scenedesmus* sp., *Phaeodactylum* sp. and *Pavlova lutheri*. Biofertilizers studies also focused on microalgae harvesting, as illustrated in the light green cluster including the *harvest*, *substrate* and *material* concepts. The algal biomass is usually harvested during the exponential growth phase to obtain maximum growth yields and a better amino acid profile in order to have an efficient biostimulant activity. The biomass was recovered by centrifugation or filtration and some studies on biofertilizers tested electrocoagulation [82]. Last, the emerging concepts of the biofertilizers database were *photobioreactor*, *carbon*, *digestate*, *microalgae biomass*, *photosynthetic*, *protein*, *agriculture*, *anaerobic*, *biogas production*, *carbohydrate*, *centrate*, *chemical*, *culture medium*, *cyanobacteria*, *digestion*, *dilution*, *matter* and *maximal nutrient removal*. These concepts only represented a maximum of three publications per year until 2016 because of the youth of this research topic but pointed out an interest for the innovative use of cyanobacteria as possible biofertilizers. Finally, Win et al. (2018) also pointed out the main limitations in the development of microalgae-based biofertilizers, namely the short shelf life of biomass and need for expensive cold storage [83].

#### 2.2.4. Nutraceuticals

**Figure 7:**
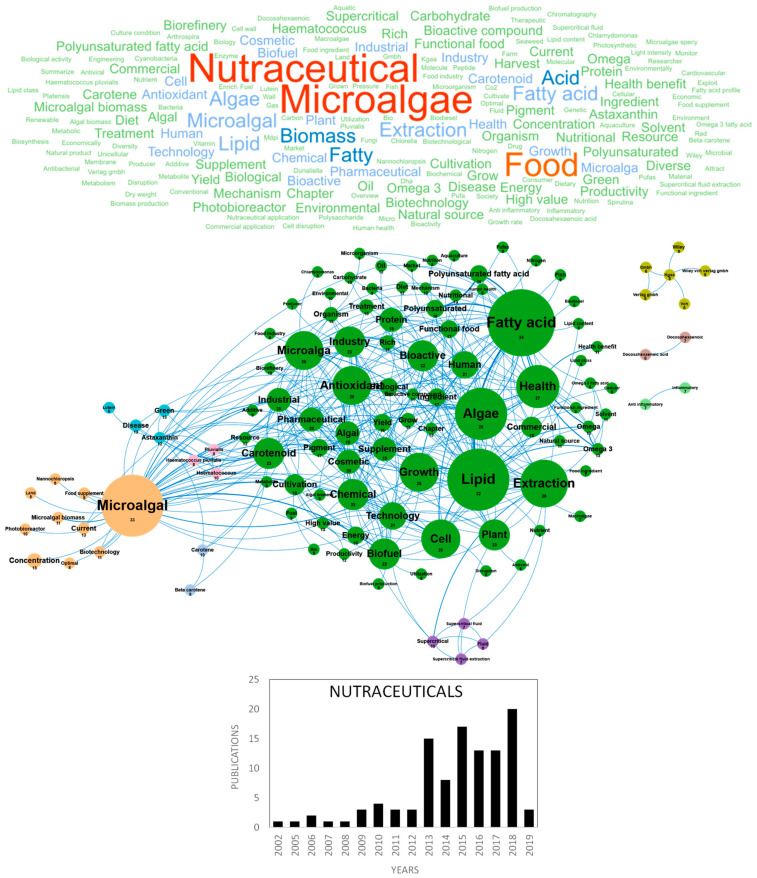
Main concepts, concepts network and annual number of the European scientific publications related to the nutraceutical field in 108 publications (400 links; 5 coocc; 5 occ; and 9 clusters).

Microalgae and cyanobacteria have been used as human food for hundreds of years; for example, Aztecs consumed Spirulina (*Arthrospira* sp.) from Lake Texcoco in Mexico around 1300 AD [86]. Today only few species (GRAS species) have obtained authorization for human food but microalgae and cyanobacteria attract the attention of researchers and consumers because they contain a wide range of nutrients and bio-active compounds, including proteins, polysaccharides, lipids, pigments, polyphenols, minerals and vitamins of high nutritional value. Consumption of whole dried microalgae and cyanobacteria has greatly increased during the last 20 years and they are now considered as an eco-friendly/organic sustainable food, alternative and complementary to “classical” food, with health-promoting properties [86]. More recently, in the 2010s, microalgae- and cyanobacteria-based food supplements or nutraceuticals started to be produced and marketed. Lipids (polyunsaturated fatty acids (PUFAs): EPA and DHA) and pigments (carotenoids) largely dominate these markets. Nutraceuticals can be defined as food nutrients that not only supplement the diet but also facilitate the prevention or treatment of diseases and disorders, according to the producers’ claims. Recent papers have reviewed the state of the art about the biotechnological production and use of microalgae [87,88], with a particular focus on their use for food and feed [89,90,91] or nutraceuticals [92,93,94,95]. In Europe, the two main countries publishing in the nutraceuticals field were Italy and Spain with 29 and 26 publications, respectively (Figure 10d). Figure 7 shows the word clouds of the main concepts contained in scientific publications dedicated to microalgae nutraceuticals, networks and clusters established between these concepts and the temporal production of these publications. The figure shows that the scientific community interest in microalgae nutraceuticals started in 2013, with about 15 publications/year. The five dominant concepts were *nutraceutical*, *microalgae*, *food*, *biomass* and *acid*. These major and ubiquitous concepts were dismissed to obtain a more detailed network of concepts, including nine clusters in Figure 7. The main cluster identified in green is dominated by the *antioxidant*, *antiviral* and *bioactive compounds* concepts and grouped all microalgae molecules and bioactive substances identified as nutraceuticals. Among the nutraceutical supplements or *functional food* of this cluster, *fatty acid*, *PUFAs*, *rich* and *carotenoids* overlook widely and their concepts networks are tightly connected. Microalgae constitute *natural sources* of *omega-3* or *PUFAs* that have unequivocally proved to improve human health and limit *diseases* (e.g., cardiovascular disorders, cancer, type 2 diabetes, inflammatory bowel disorders, asthma, arthritis, skin disorders, depression and schizophrenia). Due to a clear wild fish capture over exploitation in the last few years and their use as a raw material for oil production (specifically ω3 oils), purification of PUFAs from microalgae to produce omega-3 capsules has attracted attention since 2013, a time when fish oil and terrestrial vegetables (e.g., flaxseeds) were the only source used to obtain them.

*Carotenoids*, including *β-carotene*, astaxanthin, fucoxanthin, zeaxanthin and lutein are also valorized for a wide range of nutraceutical activities (antioxidant, prevention of DMLA) and functional properties such as food coloring in sweets, salmon, cheese, etc. *Carotenoids* research is mostly focused on the use of *astaxanthin*, *lutein* and *green* (microalgae) to cure *diseases*. The two small clusters in pink and blue highlight the niche market of *astaxanthin* produced by *Haematococcus* sp. for salmon coloring but *Chlorella* and *Spirulina* are dominating the worldwide microalgae nutraceuticals markets because of their popularity as a healthy-food in supermarkets [86]. European researchers have also studied the possible use of *carbohydrates* as microalgae-based nutraceuticals. Microalgae and cyanobacteria produce various polysaccharides with diversified structures and biological activities that are widely used in the food industry as thickeners and gelling additives. Exopolysaccharides (EPS) production from microalgae is dominated by the red microalgae genus *Porphyridium* which can be grown in different production systems and at different latitudes [96] and has multiple nutraceutical and pharmaceutical activities such as antioxidant, antitumor, antibacterial and anticoagulant activities [86,90]. The *aquaculture* concept also appeared in this cluster. Indeed, microalgae such as *Tetraselmis*, *Isochrysis*, *Pavlova*, *Phaeodactylum*, *Chaetoceros*, *Nannochloropsis*, *Skeletonema* and *Thalassiosira* are commonly used as feed for aquaculture and health-promoting supplements in aquaculture farms, reducing fish larvae mortality, stimulating immunity and limiting the use of antibiotics. Microalgae are also considered today as an interesting nutraceutical feed for ruminants, pigs, poultry and pets. Minor bioactive microalgae compounds were not highlighted in our bibliometric analysis. This may be explained by a low interest for their nutraceutical activity, small or nonexistent markets and low number of publications dealing with their nutraceutical activity. For example, chlorophylls that have demonstrated cell repair activities, antimutagenic, anticarcinogenic and antioxidant activities do not appear in a significant cluster despite their use as colorants by the food industry. The same observation could be made for phycobiliproteins [97], which are already used as natural dyes in food and considered as potent antioxidant, anti-inflammatory, neuroprotective and hepatoprotective pigments. No clusters were also identified for vitamins and sterols. Microalgae sterols [98] have diverse biological functions such as antioxidant, anti-inflammatory, antimicrobial activities and were shown to limit the progression of certain cancers and the risks of cardiovascular diseases, neurodegenerative diseases and diabetes [99]. Volatile and phenolic compounds were also absent from this analysis despite their antimicrobial and anti-inflammatory activities [41]. A cluster linked to recent research on green extraction techniques was identified through the *supercritical fluid extraction* concept. Two other small clusters highlighted niche research specific to *anti-inflammatory* drugs and *DHA* on the right of the word clouds.

From an economic point of view, the industrial orientation of microalgae scientific research towards the potential nutraceutical markets is highlighted by the *food industry*, *industrial*, *commercial* and *market* concepts. The function of these nutraceuticals is to be incorporated as *ingredient*, *additive* or *functional ingredient* in existing food products. This huge world economic market leads to the global production of 12,000 tons/year of dried *Spirulina* sp. (with a median price of 30 US$/kg) followed by *Chlorella* sp. (5000 tons/year), *Dunaliella salina* (3000 tons/year), *Aphanizomenon flos aquae*, *Haematococcus pluvalis*, *Crypthecodinium cohnii* and *Shizochytrium* [86]. From a technical point of view, the scientific research in Europe also focuses on the physiology, optimization and cultivation of microalgae through the *microalgal*, *photobioreactor*, *microalgae biomass*, *optimal*, *current* and *biotechnology* concepts in orange. *Nannochlorospis* is the model species of this cluster used in nutraceuticals (Figure 7). The scientific publications on nutraceuticals and microalgae are numerous with about a hundred publications related to *health*, *natural sources*, *bio* and *human health* concepts (Figure 7). The emerging concepts in the nutraceuticals field are focused on *Temperature*, *arable*, *bar*, *continuous*, *fucoxanthin*, *neutral*, *physiology*, *red phase*, *agricultural*, *Arthrospira platensis*, *bench scale reactor*, *biochemical composition*, *biomass yield*, *C18*, *cholesterol* and *domesticate strain*. Finally, some of these studies mention that the organoleptic properties such as taste, texture, color and odor of this microalgal biomass are potential bottlenecks to markets [86] and identify the cost of developing and obtaining a market authorization for a novel food as a strong barrier limiting the marketing of microalgae nutraceuticals.

#### 2.2.5. Pharmaceuticals

**Figure 8:**
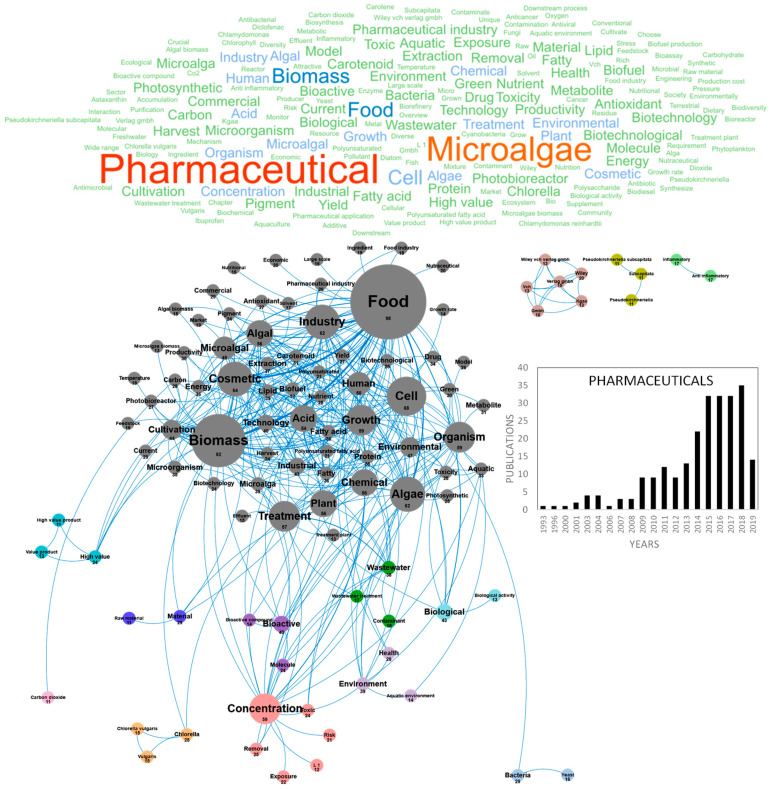
Main concepts, concepts network and annual number of the European scientific publications related to the pharmaceutic field in 239 publications (400 links; 11 coocc; 11 occ; and 14 clusters).

The potential of microalgae molecules for pharmaceutical applications has been extensively reviewed [100,101]. The application domains of microalgae metabolites for human and animal health include the prevention and improvement of various diseases including malnutrition, nutritional deficiencies, metabolic syndrome (diabetes, obesity and non-alcoholic steatohepatitis), cardio-vascular diseases, cancer, macular neurodegenerescence, cataract, inflammation, Alzheimer’s disease, depression and schizophrenia, fetal malformation, pregnancy complications, auto-immune diseases and dental diseases, as well as bacterial, parasitic and viral infections. Additionally, microalgae represent an innovative biotechnological resource for the production of high-value biomedical products such as recombinant proteins (vaccines, cytokines, hormones), bioactive polysaccharides, lipids or pigments (carotenoids, phycobiliproteins), which can be useful for the treatment, diagnosis and prevention of diseases. Microalgae have also attracted considerable interest to develop pro-cicatrizant extracts (wound healing properties), blood circulation-stimulating extracts, photosensitive pigments for phototherapy, biomaterials and biopolymers [102]. Most studies have only used in vitro assays to demonstrate the pharmaceutical potential of microalgae extracts and purified compounds, but the validation of these activities with in vivo models has become a major objective during the last five years. Following the sharp rise of scientific publications in the nutraceuticals and cosmetics fields, pharmaceutical research on microalgae has been considerably expanding since the early 2010s and the number of technical publications and reviews in this topic has now reached 239. Figure 8 shows the word clouds of the main concepts contained in the scientific publications on pharmaceuticals, networks and clusters established between these concepts and the temporal production of these publications. When the three most important concepts, *pharmaceutical*, *microalgae* and *biomass*, were dismissed from the concept network graph, a network of 14 clusters was created. The main cluster in gray highlights the interconnection of pharmaceutical concepts with the close concepts of *food* and *biomass* that dominate the market and *nutraceutical*, *nutritional* and *cosmetic*. Many molecules can be classified as nutraceuticals but the concept of *drug* establishes a clear link with the pharmaceutical domain. Within this cluster, *pigments* and *PUFAS* are highly studied for their pharmaceutical activities. PUFAs have been studied for their potential to prevent and improve cardiovascular disorders, cancer (anti-metastatic activity, antiangiogenic activity and chemo- and radiosensitization of tumor cells), type 2 diabetes, inflammatory bowel disorders, asthma, arthritis, skin disorders, depression and schizophrenia. *Pigments* and especially *carotenoids* have demonstrated important pharmacological activities, to improve fertility (astaxanthin), prevent macular degeneration (lutein and zeaxanthin), rheumatoid arthritis, cardiovascular diseases, neurodegenerative diseases, obesity (fucoxanthin) and cancers (β-Carotene, fucoxanthin, zeaxanthin, violaxanthin, lutein, alloxanthin, etc.). Because of their use as fluorescents probes in flow cytometry and microscopy and for their use in antibody conjugation methods, *phycobiliproteins* have generated important research and markets for medical diagnosis, in addition to their use in the food and cosmetic industries as natural dyes [103]. The phycobiliproteins production from microalgae using new methods is led by *Porphyridium purpureum* in the case of Phycoerythrin and by *Spirulina* for Phycocyanin production [104,105]. A specific interest was dedicated to the risk associated to microalgae *toxins* for human health, as illustrated by the pink and purple clusters through the *concentration, risk, toxic, aquatic environment* and *environment* concepts. Toxins are pharmacologically *active compounds* synthesized by microalgae (especially dinoflagellates and diatoms) and cyanobacteria, with deleterious effects for human health. Some toxins have gained importance as pharmacological tools, and could be useful to design new bioactive compounds [106]. Most studies dealing with pharmaceutical activities of microalgae molecules were carried out on *Chlorella* sp. and *Pseudokiclorella* sp. as shown in the orange cluster. In this cloud of pharmaceutical concepts, some small clusters can be differentiated. The *high value product* in blue, the *biological activity* in light blue, the *bioactive compounds* and *molecules* in purple, the *anti-inflammatory* properties in green can be considered as target concepts. This domain is also strongly associated with the *economic* and *commercial* objectives of the *pharmaceutical industry* that drives the industrial development of microalgae-based pharmaceuticals. Pharmaceuticals from microalgae have attracted the interest of researchers for many years as shown in Figure 8. Fewer than 10 publications/year were produced in the field before 2009, then 10–20 publications/year between 2009 and 2014 and more than 35 publications/year since 2015 (Figure 8). Within these 239 publications, the emerging concepts of the last three years show the trend of the research and targeted market. *Drugs* are among the emerging concepts and in particular anti-depressant and anxiolytic drugs (*amitriptyline, Clomipramine* and *behavior*). Other emergent microalgae-based pharmaceutical concepts are *culture collection*, *industrial production*, *long term*, *methyl*, *stream*, *urban wastewater*, *additivity*, *algal treatment*, *alteration*, *amitriptyline*, *basel*, *behavior*, *bioactive component*, *bubble column pbr*, *catalytic*, *classification*, *clomipramine* and *collection strain*.

#### 2.2.6. Cosmetics

**Figure 9:**
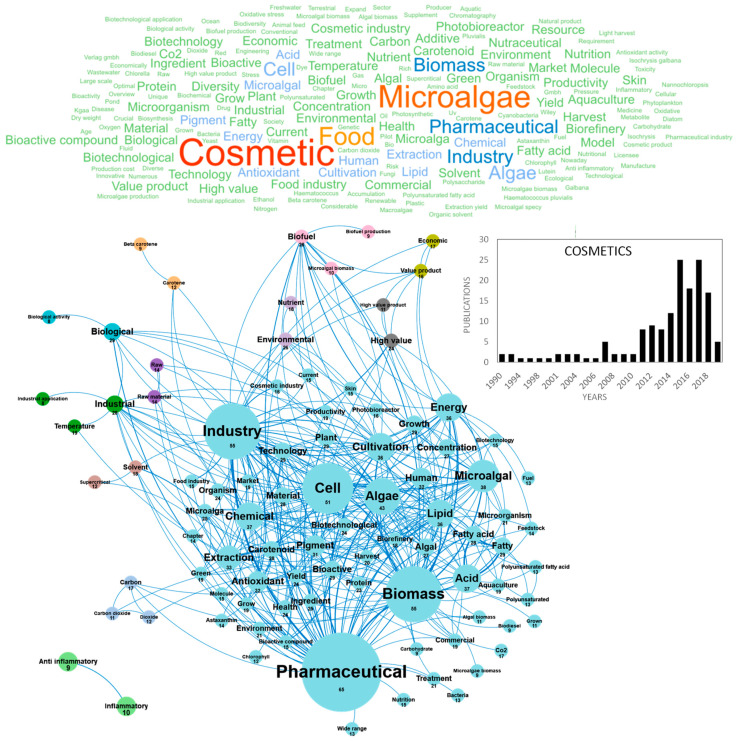
Main concepts, concepts network and annual number of the European scientific publications related to the cosmetic field in 154 publications (300 links; 8 coocc; 8 occ; and 12 clusters).

**Figure 10:**
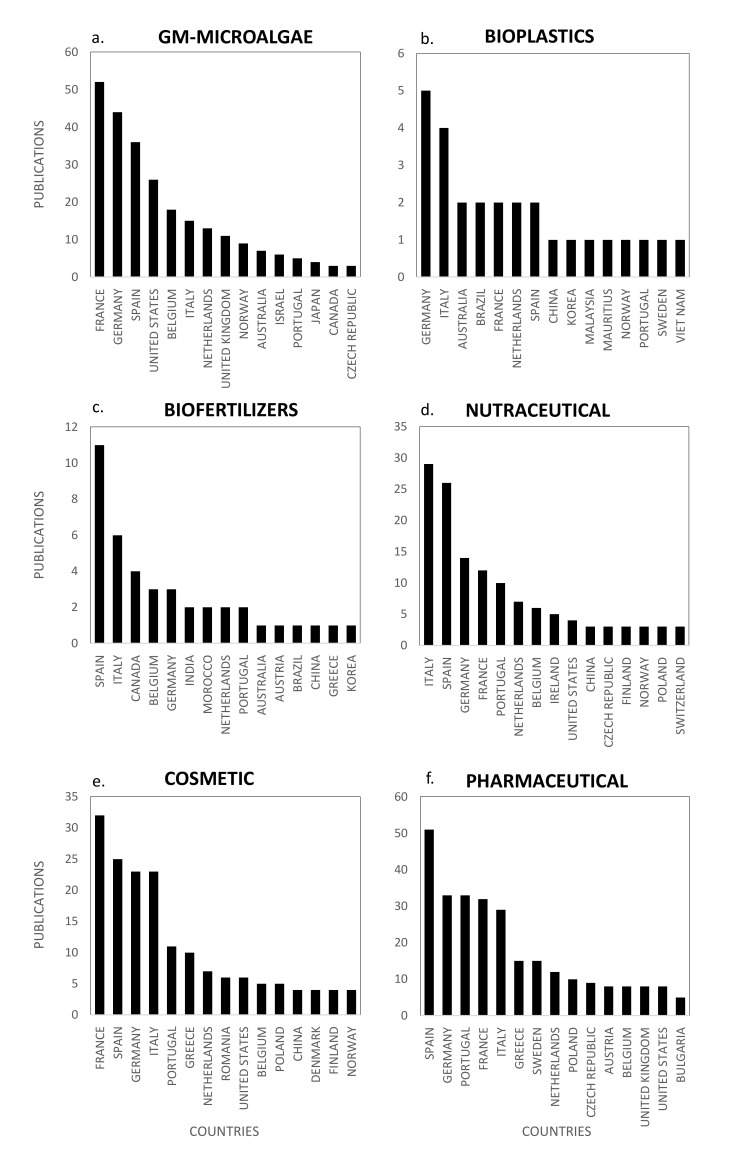
Scientific production by country for the emerging markets of: GM-microalgae (**a**); bioplastics (**b**); biofertilizers (**c**); nutraceutical (**d**); cosmetic (**e**); and pharmaceutical (**f**).

The current production of algae extracts for cosmetics is largely dominated by seaweeds and the use of microalgae was relatively confidential until the 2010s, when the societal demand for skin care and well-being treatments started to grow significantly. In response to these new markets, researchers and industrials developed relevant screening in vitro tests, assessed original microalgae species to propose innovative extracts to consumers and started to demonstrate their effective activities to improve the structure, morphology, appearance and health of the skin [107]. The market size for microalgae-based cosmetics is expected to grow significantly in the foreseeable future, because microalgae bioactive extracts are considered as relatively new and innovative as compared with seaweeds ones, and also because microalgae and cyanobacteria biodiversity is huge, offering a wide range of unexploited pigments, polysaccharides, polyphenols, with effective “cosmeceutic” activities. At the frontier between cosmetics and pharmaceutics, cosmeceutics can be defined as “*cosmetic products with biologically active ingredients claiming medical or drug-like benefits*” [107]. These include biological activities such as antioxidant, anti-aging, sunscreen and UV protective, immune protective, stress protective, moisturizing and texturizing, tanning, make-up (eyeshadows) and masking odors, whitening, stimulating blood circulation and cicatrizating. Publications reviewing and studying cosmetics from microalgae have been published since 1990, but the topic was very confidential with only one publication per year produced in Europe until 2010. A high inflection was observed in 2015, when the number of publications reached about 25 publications per year (Figure 9). The top publishers in the field were France, Spain, Germany and Italy with 24–32 papers published (Figure 10e). Figure 9 shows the word clouds of concepts contained in these scientific publications, networks and clusters established between these concepts and the temporal production of these publications. The main concepts that emerged from this cosmetic European database were *cosmetic*, *microalgae* and *food*. By dismissing these ubiquitous concepts, a network of 12 clusters was highlighted in the 154 publications. The cosmetics market was closely linked to the *pharmaceutical* application. The main cluster in blue is indeed dominated by the *pharmaceutical* concept (Figure 9). The research for *antioxidant* or *anti-inflammatory* activities is at the heart of the nutraceutical research and cosmetic markets. A focus is made on the potential of *bioactive compounds* from microalgae and their potential uses in cosmetics mainly in the treatment of *skin* problems such as aging, tanning and pigment disorders [107]. These authors listed the biological activities of microalgae and cyanobacteria in cosmetics and confirmed that most studies deal with the *antioxidant* and *anti-inflammatory* potential of microalgae extracts and molecules [107]. These cosmeceutical bioactive compounds were essentially pigments but also carbohydrates and fatty acids. Among the 154 European publications, the concepts *pigment, carotenoid, astaxanthin, chlorophyll* and *beta carotene* highlight the predominance of pigments in European studies.

Carotenoids from microalgae and cyanobacteria have demonstrated a wide range of cosmetics and cosmeceutic activities, including antioxidant, photoprotection, anti-inflammatory and anti-allergenic properties for skin [108]. Pigments were also studied for the production of natural colorants for beauty cosmetics (lipsticks with phycocyanin for eye shadows [109], tanning cosmetics containing canthaxanthin [110], eye liners, etc. *Proteins* and *carbohydrates* were also widely studied in cosmetics publications for their potential use as *ingredients* and texturizing agents. Polysaccharides have mostly been used as excipients (thickeners) and for their moisturizing actives [111]. Some methanolic extracts of exopolysaccharides were also studied for their antioxidant properties [112] and exopolysaccharides produced by *Parachlorella* improved the health and appearance of skin. EPS from microalgae has been used in commercial cosmetic products since years ago, e.g., Alguard© face cream. Current studies are focusing on their possible use in cicatrizing creams and antiviral creams. Last, microalgae lipids have been studied for skin and hair nutrition in shampoos. The applications of microalgae in cosmetic products have recently gained more attention and recent researches are focusing on original bioactives such as lycopene [113,114], canthaxanthin [110], phytohormones [115,116] and mycosporine-like amino acids as renewable bio-sourced UV filters [117]. Innovative applications are also searched for specific markets such as thalassotherapy [107] and thermal cures. No microalgae species were predominant in the European database but the skincare market was dominated by *Chlorella*, *Arthrospira*, *Nannochloropsis*, *Porphyridium*, *Nostoc* and *Dunaliella* for their potential for preventing skin aging, protecting against UV light damage, and oxidative stress [107]. From an economic point of view, this cosmetics market has a very strong link with manufacturers. The *industry, industrial, application* and *temperature* concepts are highlighted and patents are numerous, both for cosmeceutic applications and customized microalgae culture systems allowing the safe and stable on-site production of original microalgae and extracts [107]. To finish, the emerging concepts of this cosmeceutics database were identified as *g dry weight*, *red phase*, *acid production*, *aggregate*, *bar*, *behalf*, *bench scale reactor*, *bioactive component*, *compound synthesize*, *cultivation condition*, *deal*, *economy*, *extraction procedure*, *fermentation process* and *Fourier transform*.

## 3. Material and Methods

### 3.1. Building the Bibliographic Database

A bibliographic database was built through a literature search performed in February 2019 including all reports published to date. The use of the Scopus database was compulsory to obtain a format compatible with the bibliometric analysis using the Orbit Intellixir^®^ software. The keywords “microalgae” and “phytoplankton” were used to list European publications (including authors from Austria, Belgium, Bulgaria, Cyprus, Croatia, Czech Republic, Denmark, Estonia, Finland, France, Germany, Greece, Hungary, Ireland, Italy, Latvia, Lithuania, Luxembourg, Malta, Netherlands, Poland, Portugal, Romania, Slovakia, Slovenia, Spain, Sweden and UK) including at least one author from Europe. The keywords “microalga(e)” and “phytoplankton” were both selected to include environmental/ecophysiological studies as well as research and development projects dedicated to biotechnological applications for bioremediation, energy, feed, food, cosmetics, pharma, etc. We did not exclude publications dealing with cyanobacteria, considering that the research domains for these prokaryotic organisms were similar to those of microalgae.

### 3.2. Bibliometric Analysis: Data Extraction, Analysis, and Graphical Formatting

The bibliographic database was last updated in February 2019 and it contained 26,137 publications, 46,789 authors, 2393 affiliations and 423,567 concepts. As previously described [11], a “concept” designates a word (or group of words) present in the title, summary or keywords of a publication, that can be extracted and identified using a bibliometric software. The occurrence of a concept is the number of documents containing this concept, and co-occurrence the number of documents linking several concepts. The bibliographic database, including references without duplicates, was imported from Scopus into the Orbit Intellixir^®^ bibliometric software and analyzed to quantify the scientific production in a field of research per year, country. Major and emerging research concepts were graphically represented using the most relevant formats available in the Orbit Intellixir^®^ software. Data were analyzed to highlight the latest trends in research topics, identify the most explored research concepts, and highlight opportunities in the research organizations from Europe. Emerging concepts were defined as concepts that showed the greatest increase in frequency of use in the database over the last two years. A manual selection of emerging concepts was performed, as some of them were relevant for our study (e.g., name of molecules and application domains) while others were less (publisher name, etc.). A growth factor (GF) was calculated to highlight the concepts with the highest emergence over the past two years (2017–2019). GF was calculated as Equation (1).

(1) $$G=\frac{P_{2019}-P_{2017}}{P_{2017}}$$

with *P* the number of cumulative scientific publications containing the concept at one time.

## 4. Conclusions

This review analyzed 26,137 publications at the European level and gives an in-depth overview of microalgae research with potential markets applications until 2019. The main research concepts contained in the publications, the networks between these concepts, emerging concepts and the annual production of papers were highlighted. The most important scientific works in link with microalgae market applications were dedicated to six major market opportunities for microalgae: the production of biofuels, bioplastics, biofertilizers, nutraceuticals, pharmaceuticals and cosmetics. Research and developments dealing with the production of microalgae-based biofuels have attracted much interest of public and private labs, with more than 1202 papers published in Europe. These works benefited from significant public financial support that allowed to reach high levels of technology readiness. A significant market growth for algal biofuels is expected in the next years, as they hold great promise as a greener and sustainable alternative to fossil and agrofuels, but an improvement in their competitiveness is still necessary before large-scale exploitation. Research dedicated to the production of microalgae-based bioplastics or biofertilizers is still in early development, with only about 20 scientific papers published each year in Europe and related markets can be considered as niche markets with a high potential of evolution. It is most likely that these research domains are going to develop quickly in parallel to related markets, according to the high demand for eco-friendly solutions to replace petroleum-based plastics and chemical fertilizers. Last, the development and marketing of microalgae-based nutraceutics, pharmaceuticals and cosmetics has attracted much research interest with more than 600 publications for all these domains in Europe. Although research, developments and markets in these domains are much more advanced for seaweeds, microalgae offer new opportunities for original bioactive molecules and innovative extracts, with the guarantee of a regular supply independent from seasonality and easier management of production, quality and traceability. Finally, two technological domains could have a predominant influence on the marketability and competitivity of microalgae-based molecules, extracts and biomass: the possibility to transform strains to obtain GM-microalgae with high metabolic potential, and innovations in the fields of microalgae growth, harvest and extraction technologies at industrial scale. In this context, Europe, and particularly the European Atlantic Area regions, have many assets to significantly develop the microalgae bioeconomy and counterbalance the current production and market model.

## Figures and Tables

**Table 1:** Main publications cited in Europe in the domain of microalgae harvesting.

Title of Publications	Citations	References
Algal-bacterial processes for the treatment of hazardous contaminants: A review	659	[21]
Flocculation as a low-cost method for harvesting microalgae for bulk biomass production	321	[22]
Harvesting of microalgae by bio-flocculation	217	[23]
Harvesting techniques applied to microalgae: A review	189	[15]
Carbon dioxide capture from flue gases using microalgae: Engineering aspects and biorefinery concept	188	[24]
Flocculation of *Chlorella vulgaris* induced by high pH: Role of magnesium and calcium and practical implications	164	[25]
Evaluation of electro-coagulation-flocculation for harvesting marine and freshwater microalgae	133	[26]
Milking of microalgae	114	[27]
Harvesting microalgal biomass using submerged microfiltration membranes	113	[28]
Pretreatment of microalgae to improve biogas production: A review	109	[29]
Harvesting the microalgae *Phaeodactylum tricornutum* with polyaluminum chloride, aluminium sulphate, chitosan and alkalinity-induced flocculation	101	[30]
Diatom silicon biomineralization as an inspirational source of new approaches to silica production	96	[31]
Characterization of phytoplankton communities in the lower St. Lawrence Estuary using HPLC-detected pigments and cell microscopy	84	[32]
Biogenic carbon flows through the planktonic food web of the Amundsen Gulf (Arctic Ocean): A synthesis of field measurements and inverse modeling analyses	83	[33]

**Table 2:** Top 15 countries publishing on the topic of microalgae harvesting in the European database.

Countries	Publications
Spain	52
Italy	22
Belgium	20
Germany	20
France	16
Portugal	13
United States	13
Netherlands	12
Sweden	7
Australia	6
Canada	6
Denmark	6
United Kingdom	6
Austria	5
Chile	5

**Table 3:** Top 13 emerging concepts during 2017–2019 and growth factors (GF) identified in the microalgae scientific publications in the harvest European database.

Emerging Concepts	GF
*Asian*	3
*Incubation*	3
*Mitigate*	3
*Robust*	3
*Stock*	3
*Adhere*	2
*Agriculture*	2
*Aluminum sulfate*	2
*Artificial breed*	2
*Artificial substrate*	2
*Asian market*	2
*Benthic community*	2
*Blend*	2
*Breed*	2
*C16*	2
*C18*	2
*Commercial scale*	2
*Continental*	2
*Copper*	2
*Driven*	2

**Table 4:** Main publications cited in the microalgae field in Europe related to the extraction.

Title of Publications	Citations	References
Effect of temperature and nitrogen concentration on the growth and lipid content of *Nannochloropsis oculata* and *Chlorella vulgaris* for biodiesel production	647	[37]
Improved extraction of vegetable oils under high-intensity ultrasound and/or microwaves	303	[38]
Hydrothermal treatment (HTT) of microalgae: Evaluation of the process as conversion method in an algae biorefinery concept	236	[39]
Harvesting of microalgae by bio-flocculation	217	[23]
Innovative natural functional ingredients from microalgae	190	[40]
Screening for bioactive compounds from algae	183	[41]
Downstream processing of algal polyunsaturated fatty acids	158	[42]
An economic, sustainability, and energetic model of biodiesel production from microalgae	152	[36]
Biotechnological production of lutein and its applications	151	[43]
Optimization of accelerated solvent extraction of antioxidants from *Spirulina platensis* microalga	144	[44]
Lipid extraction from the microalga *Phaeodactylum tricornutum*	131	[45]
Freshwater phytoplankton quantification by chlorophyll a: A comparative study of in vitro, in vivo and in situ methods	127	[46]
“Solvent-free” ultrasound-assisted extraction of lipids from fresh microalgae cells: A green, clean and scalable process	123	[35]

**Table 5:** Top 15 countries publishing on the topic of microalgae extraction in the European database.

Countries	Publications
Spain	120
France	88
Italy	63
Germany	62
Netherlands	37
Portugal	27
Sweden	19
United Kingdom	19
Denmark	15
Belgium	14
Poland	13
United States	13
Ireland	10
Brazil	9
China	9

**Table 6:** Top 13 emerging concepts during 2017–2019 and growth factors (GF) identified in the microalgae scientific publications in the extraction European database.

Emerging Concepts	GF
*Springer nature*	8
*Anthropogenic*	4
*Biomolecule*	4
*Germany*	4
*Np*	4
*Prokaryotic*	4
*Springer verlag gmbh*	4
*Springer verlag gmbh germany*	4
*Carotenoid extraction*	3
*Enzymatic treatment*	3
*Evolve*	3
*Figure*	3
*Ribosomal*	3
*See fulltext*	3
*Universal*	3
*Ag*	2
*Agriculture*	2
*Alkyl*	2
*Allocation*	2
*Barcod*	2
